# Unveiling mungbean yellow mosaic virus: molecular insights and infectivity validation in mung bean (*Vigna radiata*) via infectious clones

**DOI:** 10.3389/fpls.2024.1401526

**Published:** 2024-08-02

**Authors:** Madhumitha Balasubramaniam, Tamilnayagan Thangavel, Karupiah Eraivan Arutkani Aiyanathan, Sakthi Ambothi Rathnasamy, Veera Ranjani Rajagopalan, Mohankumar Subbarayalu, Senthil Natesan, Selvaraju Kanagarajan, Raveendran Muthurajan, Sudha Manickam

**Affiliations:** ^1^ Department of Plant Pathology, Agricultural College and Research Institute, Tamil Nadu Agricultural University, Madurai, Tamil Nadu, India; ^2^ School of Agricultural Sciences, Karunya Institute of Technology and Sciences, Coimbatore, Tamil Nadu, India; ^3^ Department of Agricultural Entomology, Tamil Nadu Agricultural University, Coimbatore, Tamil Nadu, India; ^4^ Department of Biotechnology, Center for Plant Molecular Biology and Biotechnology, Tamil Nadu Agricultural University, Coimbatore, Tamil Nadu, India; ^5^ Department of Plant Breeding, Swedish University of Agricultural Sciences, Lomma, Sweden; ^6^ School of Science and Technology, The Life Science Centre, Örebro University, Örebro, Sweden

**Keywords:** mungbean yellow mosaic virus, cloning, yellow mosaic disease, agroinoculation, phylogeny, recombinant analysis

## Abstract

Yellow mosaic disease (YMD) with typical symptoms of alternating bright yellow to green patches associated with stunting, downward cupping, and wrinkling has been observed in mung bean on agricultural farms in Coimbatore, Tamil Nadu, India. PCR using gene-specific primers indicated the presence of the yellow mosaic virus in symptomatic plants. Rolling circle amplification (RCA) followed by restriction digestion detected ~2.7 kb of DNA-A and DNA-B, allowing the identification of a bipartite genome. The full-length genome sequences were deposited in NCBI GenBank with the accession numbers MK317961 (DNA-A) and MK317962 (DNA-B). Sequence analysis of DNA-A showed the highest sequence identity of 98.39% to the DNA-A of mungbean yellow mosaic virus (MYMV)-Vigna radiata (MW736047), while DNA-B exhibited the highest level of identity (98.21%) to the MYMV-Vigna aconitifolia isolate (DQ865203) reported from Tamil Nadu. Recombinant analysis revealed distinct evidence of recombinant breakpoints of DNA-A within the region encoding the open reading frame (ORF) AC2 (transcription activation protein), with the major parent identified as MYMV-PA1 (KC9111717) and the potential minor parent as MYMV-Namakkal (DQ86520.1). Interestingly, a recombination event in the common region (CR) of DNA-B, which encodes the nuclear shuttle protein and the movement protein, was detected. MYMIV-M120 (FM202447) and MYMV-Vigna (AJ132574) were identified as the event’s major and minor parents, respectively. This large variation in DNA-B led us to suspect a recombination in DNA-B. Dimeric MYMV infectious clones were constructed, and the infectivity was confirmed through agroinoculation. In future prospects, unless relying on screening using whiteflies, breeders and plant pathologists can readily use this agroinoculation procedure to identify resistant and susceptible cultivars to YMD.

## 1 Introduction

Yellow mosaic disease (YMD) in legumes is one of the important constraints for pulse production in India (Varma and Malathi, 2003). Significant pulse crops such as black gram (*Vigna mungo*), moth bean (*Vigna aconitifolia*), lima bean (*Phaseolus lunatus*), pigeon pea (*Cajanus cajan*), French bean (*Phaseolus vulgaris*), cowpea (*Vigna unguiculata*), dolichos (*Lablab purpureus*), horse gram (*Macrotyloma uniflorum*), and soybean (*Glycine max*) are widely impacted by YMD infection (Ramesh et al., 2017). YMD-infected plants generally exhibit symptoms in the form of alternating green and yellow mosaic patterns on the leaves, followed by necrosis, internode shortening, and severe stunting of plants with malformed pods that contain tiny, immature, and shriveled seeds, with yield losses ranging from 5% to 100% (Rathi, 2002; Habib et al., 2007). In parts of South and Southeast Asia, four separate bipartite begomoviruses (yellow mosaic viruses, YMVs) have been reported as the cause of YMD in legumes, i.e., mungbean yellow mosaic virus (MYMV), mungbean yellow mosaic India virus (MYMIV), horse gram yellow mosaic virus (HgYMV), and *Dolichos* yellow mosaic virus (DoYMV) (Padidam et al., 1999). In India, MYMV and MYMIV are the most important YMVs, infecting many legumes. Interestingly, MYMV is most prevalent in the southern and western regions (Karthikeyan et al., 2004; Girish and Usha, 2005; Fauquet et al., 2008), whereas MYMIV is predominant in the northern, central, and eastern regions (Usharani et al., 2004). However, recently, MYMIV has been reported in South India, while MYMV has been reported in North India (Reddy et al., 2015). YMV belongs to the genus *Begomovirus*, under the family Geminiviridae, with twin geminate particles having rolling circle replication. Whiteflies (*Bemisia tabaci*) transmit the deadly begomoviruses that infect both leguminous and non-leguminous crops (Agnihotri et al., 2019; Ansar et al., 2021). The MYMV genome is characterized by a small, circular single-strand DNA with a bipartite (DNA-A and DNA-B) genome of ~2.7 kb in size with several open reading frames (ORFs). These two components share a 200-bp-long highly conserved common region (CR), which has an origin of replication (ori) and a highly conserved stem–loop or hairpin structure with the replication-assisting invariant non-nucleotide motif (TAATATTAC). Seven ORFs or genes in DNA-A (two in the virion sense and five in the complementary sense) encode the proteins needed for transcription, replication, and encapsidation. On the other hand, the DNA-B component has two ORFs in each virion sense and a complementary sense that encodes the proteins necessary for the inter- and intracellular movement of the virus (Briddon and Stanley, 2006). The development of mung bean cultivars resistant to YMD has long been considered an effective and economical method of managing MYMV (Karthikeyan et al., 2011). Previous reports have indicated that the development of resistant cultivars is substantial due to the rapid evolution of novel MYMV isolates and the complexity of the control mechanism (Akhtar et al., 2011). Therefore, an accurate description of the virus is necessary for a successful resistance breeding approach, with virus resistance screened out in the available germplasm. Field-level screening to identify resistant germplasm often fails due to the inefficacy of whitefly transmission (Sudha et al., 2013). Hence, the “agroinoculation” technique, a different approach that uses the Ti plasmid of the *Agrobacterium* for viral infection, has become superior to other phenotyping techniques in terms of infection pattern accuracy and the speed of identification of both resistant and susceptible cultivars (Sudha et al., 2013; Madhumitha et al., 2022). The present study aimed to characterize the complete genome of the MYMV isolates from mung bean in southern India. Nucleotide identity, phylogenetic relationship, and recombination analyses were assessed with those of previously reported Old World and New World begomoviruses, particularly legumoviruses, in order to identify the distinctiveness of the genome. In addition, cloning of MYMV in tandem orientation and infectivity screening using agroinoculation were performed, which could be used as additional tools for the screening of resistant lines against YMD.

## 2 Materials and methods

### 2.1 Viral source

Mung bean (*Vigna radiata* L. Wilczek) plants showing characteristic yellow mosaic, downward cupping, wrinkling, necrosis, and stunted-like YMD symptoms were collected from fields located in southern India (11°00′21.00′′ N, 76°49′24.59′′ E), Thondamuthur, Coimbatore District, Tamil Nadu, India.

### 2.2 DNA extraction from virally infected samples

The total genomic DNA was extracted using a modified CTAB method (Sudha, 2009). The preliminary virus detection methods were carried out using MYMV-specific primers, i.e., coat protein (CP) and movement protein (MP) primers (Maheshwari et al., 2014).

### 2.3 Rolling circle amplification and cloning of the full-length viral genome

The DNA samples that were PCR-positive with the MYMV gene-specific primers were subjected to rolling circle amplification (RCA) using *phi*29 DNA polymerase (TempliPhi™ 100 Amplification Kit; Merck, Darmstadt, Germany) as per the manufacturer’s protocol. The replicative DNA concatemers obtained using RCA were subjected to restriction digestion with different endonucleases, i.e., *Bam*HI, *Hind*III, *Pst*I, *Xba*I, *Eco*RI, and *Kpn*I (Thermo Scientific FastDigest, Waltham, MA, USA) for the full-length viral genomic DNA components (~2.7 kb). The linearized restricted products (~2.7 kb) were further purified, and the fragments were cloned into the cloning vector pUC18 and transformed into competent cells of the DH5α strain of *Escherichia coli*. Recombinant plasmids holding full-length genomic fragments were identified. Initially, partial sequencing was performed, and the selected clones were then sequenced through a commercial facility using the primer walking strategy at Bioserve Biotechnologies, Pvt. Ltd., Hyderabad, India.

### 2.4 Sequencing and sequence analysis

Database searches were carried out with NCBI BLAST (https://www.ncbi.nlm.nih.gov/) to align our viral genome sequences with those of previously reported viruses. The complete genome sequences of both DNA-A and DNA-B were analyzed using BioEdit version 7.0.9 (Hall, 1999). The ORFs were predicted using GENERUNNER (http://www.generunner.net/). The isolate in the present study was compared with the Old World and New World begomovirus sequences retrieved from the NCBI database. The complete nucleotide sequences of the full-length genomes were aligned using MUSCLE (Edgar, 2004), and the percentage pairwise nucleotide identity plot was generated using the SDTV 1.12 program (http://web.cbio.uct.ac.za/~brejnev/) (Muhire et al., 2014). A phylogenetic tree was constructed by aligning the selected full-length sequences of DNA-A and DNA-B through ClustalW (Thompson et al., 1994), and the tree was prepared using MEGA7 version 7.0.26 (Kumar et al., 2016). Bootstrap analysis with 1,000 replications was performed using the neighbor-joining method. Identification of the recombination events of MYMV DNA-A and DNA-B was analyzed using the Recombination Detection Program (RDP 4.97), which utilizes seven different algorithms: RDP, BOOTSCAN, GENECONV, MAXCHI, CHIMAERA, SISCAN, and 3SEQURE (Smith, 1992; Salminen et al., 1995; Padidam et al., 1999; Gibbs et al., 2000; Posada and Crandall, 2001; Boni et al., 2007; Martin et al., 2015). A cutoff of 0.05 was used as the *p*-value with default settings.

### 2.5 Construction of agroinfectious partial tandem repeat constructs and their infectivity

For the construction of infectious clones, partial tandem repeat constructs of DNA-A and DNA-B were prepared by directional cloning of a bitmer (a part of the genome with the ori), followed by further cloning of the fullmer (~2.7 kb full-length genome) of DNA-A or DNA-B. For bitmer release into DNA-A, the pUC18 plasmid (harboring the full-length 2.7-kb fragment of DNA-A) was restricted with the restriction endonucleases *Hind*III and *Pst*I. Approximately 2.4 kb, consisting of the ori, was eluted from the agarose gel and ligated into the binary vector pCAMBIA2300, restricted with the same restriction endonucleases. Recombinant colonies were screened and designated as pCamA~0.8 mer. Subsequently, one selected positive clone in the pUC18 plasmid was carried for incorporation of the full-length DNA-A genome, which was restricted with the restriction endonucleases *Hind*III/*Bgl*I and was ligated into the pCamA~0.8 mer by linearizing them through *Hind*III. Similarly, the bitmer release in DNA-B from the full-length clone in the pUC18 vector was prepared using the restriction endonucleases *Hind*III and *Sma*I. The fragment was eluted and ligated into the *Hind*III/*Sma*I-restricted pCAMBIA2300 (binary vector). The recombinant plasmid was designated as pCamB~0.4 mer. Incorporation of the full length (2.7 kb) of DNA-B from the PUC18 plasmid restriction was performed with the *Hind*III/*Pvu*II enzymes. The pCamB~0.4 mer was linearized with *Hind*III, and the full-length (2.7 kb) genome was incorporated into it. The transformation was carried out for both the DNA-A and DNA-B components with antibiotic selection. The orientation of the construct was determined through restriction analysis for both pCamA~0.8 mer, pCamB~0.4 mer, and their respective fullmer using the enzymes *Hind*III, *Pvu*II, *Sma*I, *Bgl*I, and *Pst*I.

The tandem repeat constructs of the viruses were mobilized from the *E. coli* strain XL1-Blue into the *Agrobacterium tumefaciens* strain LBA 4404 (Hood et al., 1993) using pRK 2013 as a helper plasmid in a triparental mating system (Ditta et al., 1980). The distinct colonies that appeared were confirmed transconjugants through colony PCR using internal primers specific to the DNA-A and DNA-B genomes. *Agrobacterium* harboring the viral DNA components was grown in Luria–Bertani (LB) broth at 28°C with shaking at 200 rpm. The overnight-grown lawn of bacterial growth was collected and resuspended in *Agrobacterium* (AB) minimal medium (OD_600_ = 0.8) and used for agroinoculation. The *Agrobacterium* cultures containing the constructs of DNA-A and DNA-B were mixed in equal proportion, and 30 μl of the culture was inoculated on 2-day-old sprouted seeds of 20 cultivars of mung bean [i.e., VGGRU2, VGGRU3, VGGRU4, CO8, CO7, CO980, PUSA-118, PUSA-9871, VBN (Gg)2, PLS-316, LM469, Bapatla, VGGRU1, NDM 5–3, AVT/RMI-6/1, Maduramoong, SML119, PUSA-0672, PUSA-101, and VRM (Gg)1] after puncturing the hypocotyl region with a 30-G needle using the seed-sprout method (Mandal et al., 1997). Infection was carried out at 25°C for 12 h in the dark. Subsequently, the seedlings were washed with sterile single-distilled water and sown in soil/compost (1:1) in a controlled growth chamber at 25°C with a proper relative humidity of 60%–70% and a photoperiod of 16/18 h. Hoagland’s solution was sprayed twice a week for the proper growth and development of plants. The uninoculated plants without agroinoculation were maintained as controls. The observation of symptoms in the trifoliate leaves was recorded after the 15th day of inoculation. The plants were identified as susceptible or resistant based on the presence or absence of yellow mosaic symptoms at a given time across replications. Scaling was conducted based on the rating (Singh et al., 1992). Leaves with typical YMD symptoms were collected 15–17 days after inoculation for DNA analysis and were confirmed through PCR using the CP primers.

## 3 Results

**Figure 1:**
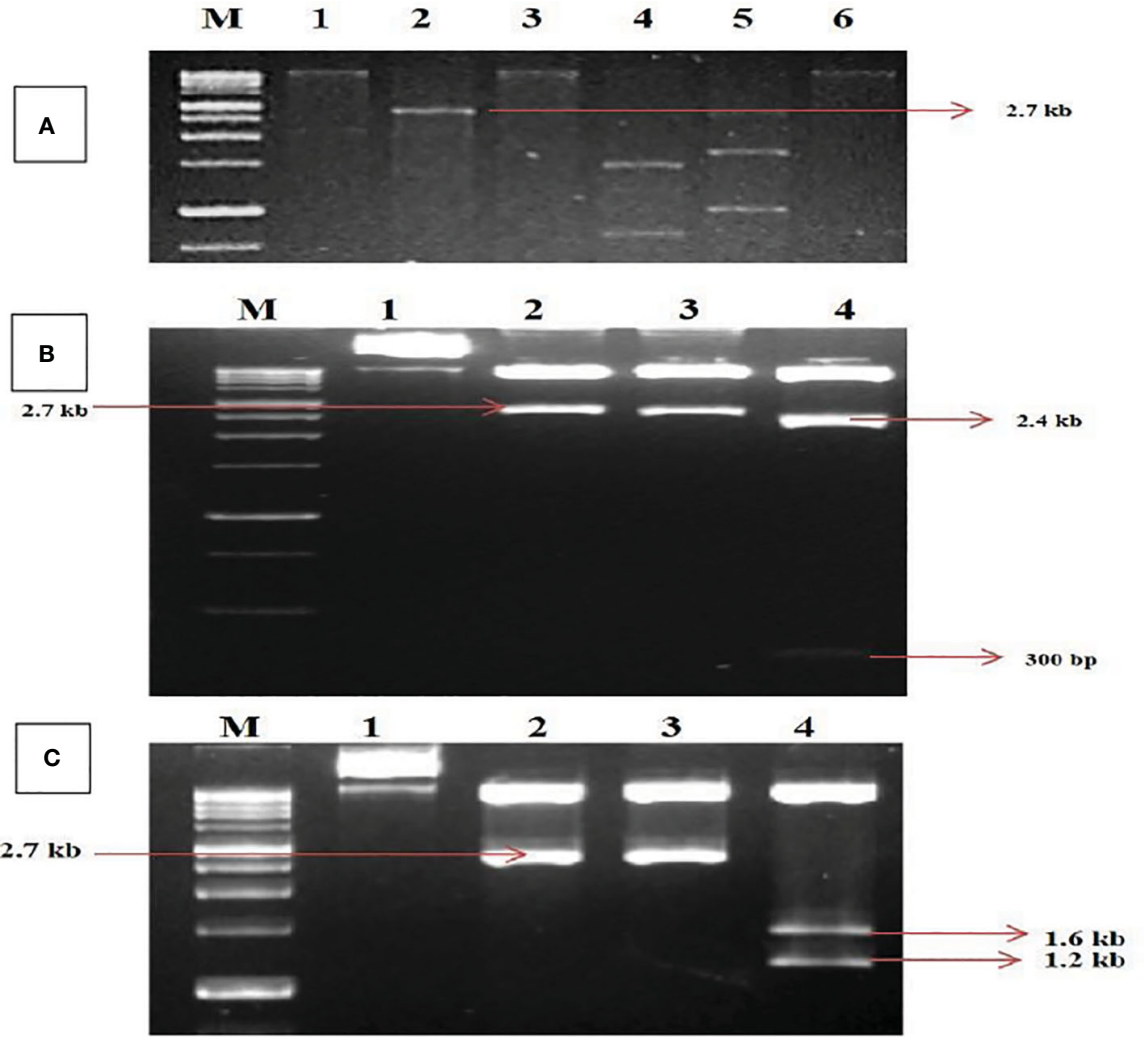
Identification and cloning of MYMV genome on tandem orientation. **(A)** Rolling circle amplification followed by restriction digestion. Lanes M (1Kb Marker),1, 2, 3, 4, 5, 6 represent digestion with *Bam*HI, *Hind*III, *Pst*I, *Xba*I, *EcoR*I and *Kpn*I respectively. **(B)** Confirmation of tandem orientation of PcamA~ 0.8 mer and full length using two different restriction endonucleases with *Hind*III and *Pst*I, Lane M - 1Kb Marker,1,2,3,4 represent Uncut plasmid DNA, a restricted product of plasmid with *Hind*III, a restricted product of plasmid with *Pst*I, a restricted product of plasmid with *Hind*III and *Pst*I. **(C)** Confirmation of tandem orientation of PcamB~ 0.4 and full length using two different restriction endonucleases with *Hind*III and *Sma*I, Lane M - 1Kb Marker,1, 2, 3, 4 represent Uncut plasmid DNA, restricted product of plasmid with *Hind*III, *Sma*I restricted product, *Hind*III and *Sma*I restricted product.

The digested RCA products revealed a single band of size ~2.7 kb on restriction with *Hind*III and *Pst*I. In comparison, *Hind*III was found to be a unique cutter in the viral genome for both DNA-A and DNA-B (**Figure 1A**) and was further utilized for the construction of infectious clones. The ~2.7-kb band of digested products was cloned. Five transformed clones (~2.7 kb) were identified and partially sequenced for DNA-A and DNA-B. The sequence analysis for obtaining potential ORFs using ORF Finder (http://www.ncbi.nlm.nih.gov/gorf/gorf.html) revealed three clones that had an arrangement of genes typical of DNA-A components.

In contrast, two clones had an arrangement typical of DNA-B components of bipartite begomoviruses, and no satellite DNA was observed. The complete nucleotide sequence for one of each clone of DNA-A and DNA-B was determined in both orientations through primer walking. The nucleotide sequences of the DNA-A and DNA-B components were determined to be 2,726 and 2,739 bp, respectively. The sequence data obtained from the two clones were submitted to the GenBank database with accession numbers MK317961 (DNA-A) and MK317962 (DNA-B). The DNA sequence studied was typical of the Old World begomoviruses, with two ORFs on the viral sense strand and four ORFs on the complementary sense strand. The viral strand consists of the ORF AV1 (~28 kDa)–CP, which overlaps with the small ORF AV2–pre-coat protein (~13 kDa), while the complementary sense strand consists of the ORF AC1–replication initiator protein (~40 kDa), ORF AC2–transcription activation protein (~15 kDa), ORF AC3–replication enhancer protein (~15 kDa), and ORF AC4–symptom determinant (~11 kDa). The DNA-B components were determined to have a genome length of 2,739 bp that encodes two ORFs: BV1–nuclear shuttle protein (~28 kDa) on the viral sense strand and BC1–MP (~33 kDa) on the complementary sense strand (**Supplementary Tables S1, S2**). For further characterization, the clones were designated as MK317961-MYMV-ThC03 and MK317962-MYMV-ThC15. For infectious clone construction, the full-length clones were subjected to tandem orientation cloning, and the orientation of the construct was determined through restriction analysis (**Figures 1B, C**).

### 3.1 Genome characterization of DNA-A

**Figure 2:**
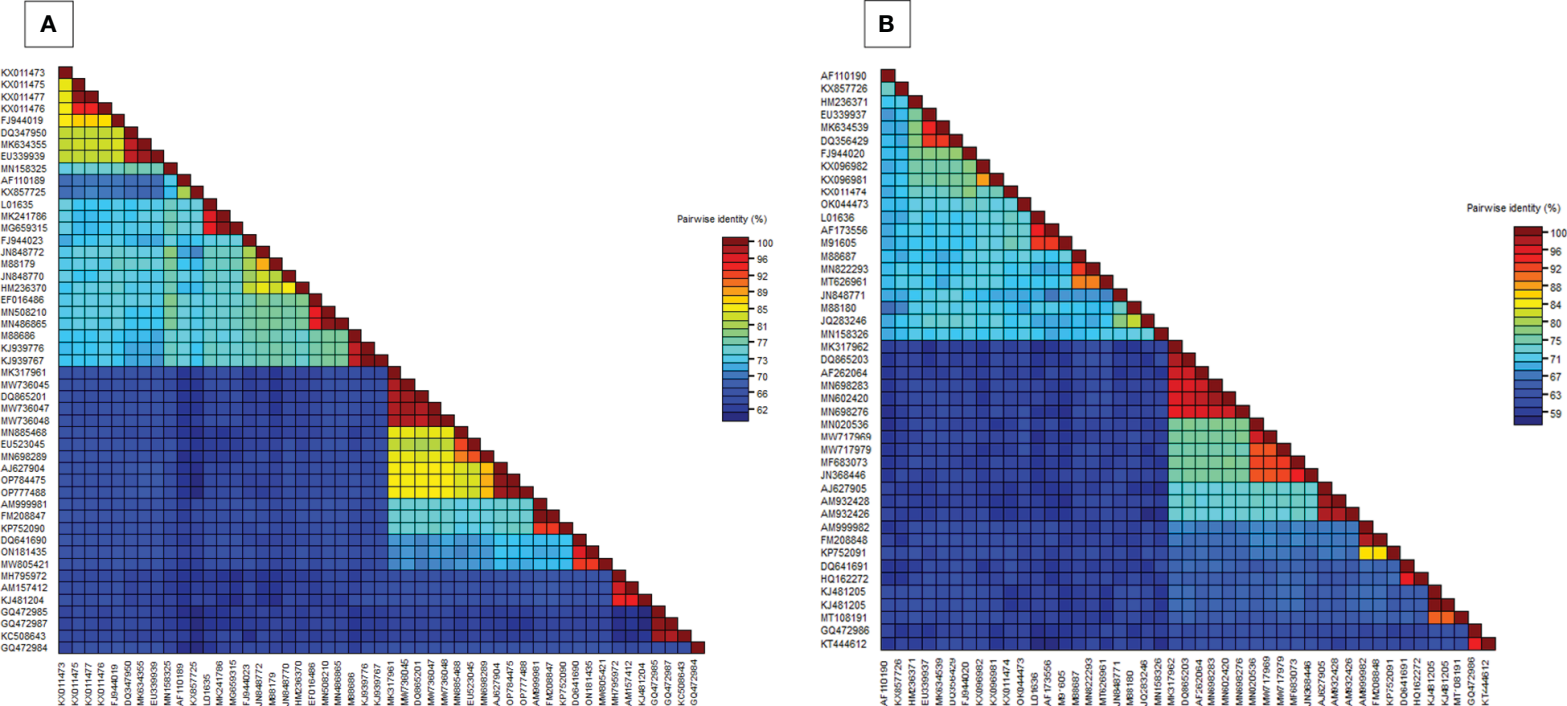
**(A)** Pairwise nucleotide identity matrices of DNA-A (MK317961) with previously worldwide reported begomovirus nucleotide sequences, **(B)** Pairwise nucleotide identity matrices of DNA-B (MK317962) with previously worldwide reported begomovirus nucleotide sequences.

**Figure 3:**
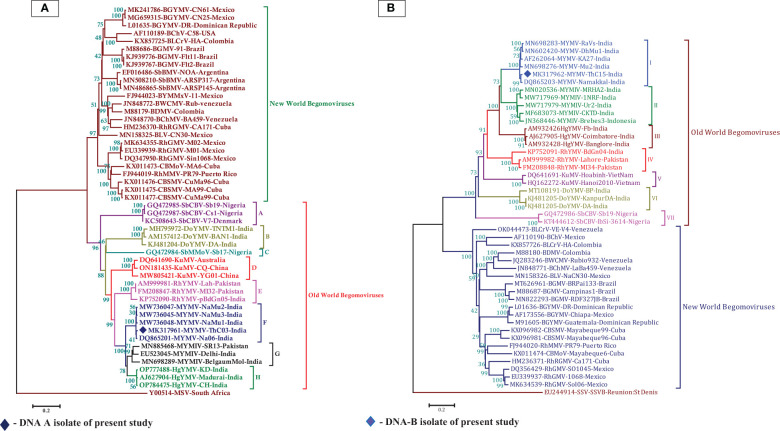
**(A)** Phylogenetic relationship between MYMV isolate with other Old World and New World begomoviruses generated from aligned full-length genome sequences of DNA-A, blue-filled diamond representing MK317961 (DNA-A) investigated in the present study, **(B)** The phylogenetic relationship between MYMV isolate with other Old World and New World begomoviruses generated from aligned full-length genome sequences of DNA-B, the blue filled diamond representing MK317962 (DNA-B) investigated in the present study. The tree was generated by the neighbor-joining method by aligning the sequences in MEGA 7 using CLUSTAL W. Vertical branches are arbitrary; horizontal branches are proportional to calculated mutation distance values at nodes indicated percentage bootstraps values (1000 replicates). mungbean yellow mosaic virus (MYMV), mungbean yellow mosaic India virus (MYMIV), dolichos yellow mosaic virus (DoYMV), kudzu mosaic virus (KuMV), horsegram yellow mosaic virus (HgYMV), rhynchosia yellow mosaic virus (RhYMV), soybean chlorotic blotch virus (SbCBV), rhynchosia rugose golden mosaic virus (RhRGMV), rhynchosia mild mosaic virus (RhMMV), rhynchosia golden mosaic virus (RhGMV), bean calico mosaic virus (BChV), common bean severe mosaic virus (CBSMV), common bean mottle virus (CBMoV), bean golden mosaic virus (BGMV), bean chlorosis virus (BChMV), bean dwarf mosaic virus (BDMV), bean white chlorotic mosaic virus (BWCMV), bean leaf crumple virus (BLCrV), bean latent virus (BLV), bean golden yellow mosaic virus (BGYMV), bean yellow mosaic Mexico virus (BYMMxV), maize streak virus (MSV), soybean mild mottle virus (SbMMoV), soybean blistering mosaic virus (SbBMV), sugarcane streak virus (SSV).

In the BLASTn search, the complete nucleotide sequence of DNA-A showed the highest sequence identity of 98.39% to the DNA-A of the MYMV-Vigna radiata (MYMV : MW736047) reported from Namakkal District, Tamil Nadu, India. Pairwise sequence identity of the isolate in the present study, DNA-A MK317961-MYMV-ThC03, with other selected Old World and New World begomoviruses, particularly legumoviruses in the BLASTn search, was also determined using the Sequence Demarcation Tool (SDT v1.2), which exhibited that the DNA-A of the Old World begomoviruses shared 98.2% - 98.3% identity with the MYMV reported from Tamil Nadu, India (MW736047, MW736048, MW736045, and DQ865201), followed by 84.2%–84.7% identity with HgYMV (AJ627904, OP784475, and OP777488), 80%–83.5% with MYMIV (MN885468, MN698289, and EU523045), 74%–74.6% with the *Rhynchosia* yellow mosaic virus (RhYMV) (AM999981, FM208847, and KP752090), 68.6%–69.3% with Kudzu mosaic virus (KuMV) (DQ641690, ON181435, and MW805421), 61.6%–62.3% with DoYMV (MH795972, AM157412, and KJ481204), 61.9% with the soybean mild mottle virus (SbMMoV) (GQ472984), and 58.3%–58.8% identity with the soybean chlorotic blotch virus (SbCBV) (GQ472985, GQ472987, and KC508643) (**Figure 2A**). Similarly, the New World begomovirus shared the highest identity of 58.3% with the common bean severe mosaic virus (CBSMV) (KX011477) from Cuba and the lowest identity of 52.9% with the beet chlorosis virus (BChV) range from the USA. The amino acid sequence of an Old World begomovirus, MYMV-DQ865201-Na06-moth bean-Indian isolate, showed 98.3%, 94.8%, 90.2%, 96.9%, and 100% sequence identity in the AC1, AC2, AC3, AC4, AV1, and AV2 ORFs, respectively. AV1 was found to be highly conserved among the six ORF proteins, with a mean value of 85% in Old World begomoviruses, followed by the AC1 protein with a mean value of 78% in amino acid levels (**Supplementary Tables S3, S4**). Based on the complete nucleotide sequence alignment of the MYMV DNA-A isolate of MYMV-MK31791-ThCo3 and 48 other published nucleotide sequences of the New World and Old World begomoviruses, infected host plant sequences were retrieved from the NCBI database. An outgroup of maize streak virus (MSV) was used for phylogenetic analysis. The phylogenetic analysis exhibited two main groups that were separated and diverged in the tree analysis. The separation of the New World begomoviruses from the Old World viruses was very well evident in this analysis. The first major group included all the New World begomoviruses that were diverged, closely related to each other, and placed in the first major branch (i.e., BGYMV, BChV, BLCrV, BGMV, SbBMV, BYMMxV, BWCMV, BDMV, BChMV, RhRGMV, BLV, RhGMV, CBMoV, RhMMV, and CBSMV), except for the Old World begomovirus. The isolate in the present study, DNA-A MYMV-MK31791-ThCo3, was placed in the second major group of Old World begomoviruses in clade F. They are closely related to the MYMV-Namakkal isolates, with the following accession numbers: DQ865201, MW736045, MW736047, and MW736048 (**Figure 3A**). The non-coding intergenic region between the ORFs AV1 and AC1 of DNA-A was found to be ~414 bp in length. Pairwise alignment of the non-coding region between ORFs AC1/AV2 in DNA-A was carried out to identify the highly conserved CR between DNA-As. The length of the deduced CR was approximately 142 nt between the cognate DNA-A and DNA-B. The CR encompasses the predicted stem and loop structure with the conserved nonanucleotide sequence TAATATTAC, which represents the origin of replication. The putative Rep-binding iteron sequence, which consists of eight nucleotide repeats, was identified to be ATCGGTGT, which occurs as one copy in the 5′ of the CR and as two tandem repeats upstream of the TATA box in DNA-A.

### 3.2 Genome characterization of DNA-B

BLASTn search analysis of the DNA-B nucleotide sequence revealed the highest sequence identity (98.21%) to the other MYMV-Vigna aconitifolia (DQ865203) reported from Namakkal District, Tamil Nadu, India. The pairwise sequence identity of the isolate in the present study, DNA-B MK317962-MYMV-ThC15, to the other Old World begomovirus isolates published in NCBI and the New World begomovirus isolates that appeared in the BLASTn search was determined using the Sequence Demarcation Tool (SDT v1.2). Analysis of the pairwise sequence identity of DNA-B exhibited 95.4%–98.1% identity to the MYMV isolates (DQ865203 and AF262064) from Tamil Nadu, India, followed by 70.2%–70.5% identity to DNA-B of MYMIV, 65.70% - 66.50% to HgYMV (AJ627905, AM932428, and AM932426), 55.9%–57.2% to RhYMV (AM999982, FM208848, and KP752091), 53.90%–54.70% identity to KuMV (DQ641691 and HQ162272), 48.40%–49.80% identity to DoYMV (MT108191 and KJ481205), and 45%–45.3% identity to SbCBV (KT444612 and GQ472986) (**Figure 2B** **).** The amino acid sequences showed the highest sequence identity of 100% to both nuclear shuttle and movement proteins of MYMV DNA-B (MN602420), followed by 100% (MP) and 99.6% (NSP) identity to MYMV DNA-B (DQ865203), 99.6% (NSP) and 100% (MP) identity to MYMV DNA-B (MN698276), and 99.6% (NSP) and 98.4% (MP) identity to MYMV DNA-B (AF262064) (**Supplementary Tables S5, S6**).

Phylogenetic analysis of the isolate in the present study (MK317962-MYMV-ThC15) was performed with 46 DNA-B sequences, which were retrieved from the NCBI database, of which 23 nucleotide sequences were from Old World begomoviruses and 21 were from New World begomoviruses. An outgroup of Sugarcane streak virus (SSV) was used in the analysis. The phylogenetic distribution showed that two main groups diverged in the tree analysis. This indicates that the Old World begomovirus isolates were placed into the first major group and the New World begomovirus isolates were grouped into the second main branch. The isolate in the present study, DNA-B MK317962-MYMV-ThC15, was grouped in clade I of the first major branch, which is closely related to the other MYMV isolates (MN698283, MN602420, AF262064, and DQ865203) reported from Tamil Nadu, India (**Figure 3B**). In the case of the DNA-B component, the non-coding intergenic region is ~986 bp in length. In the case of DNA-B, one of the tandem repeats of iteron is ATTGGTGT. In the CR, TATA motifs were seen at nucleotide coordinates 2,681–2,684. The divergence between DNA-A and DNA-B included 22 deletions in DNA-A with respect to DNA-B and 12 SNPs between DNA-A and DNA-B.

### 3.3 Recombinant analysis

**Figure 4:**
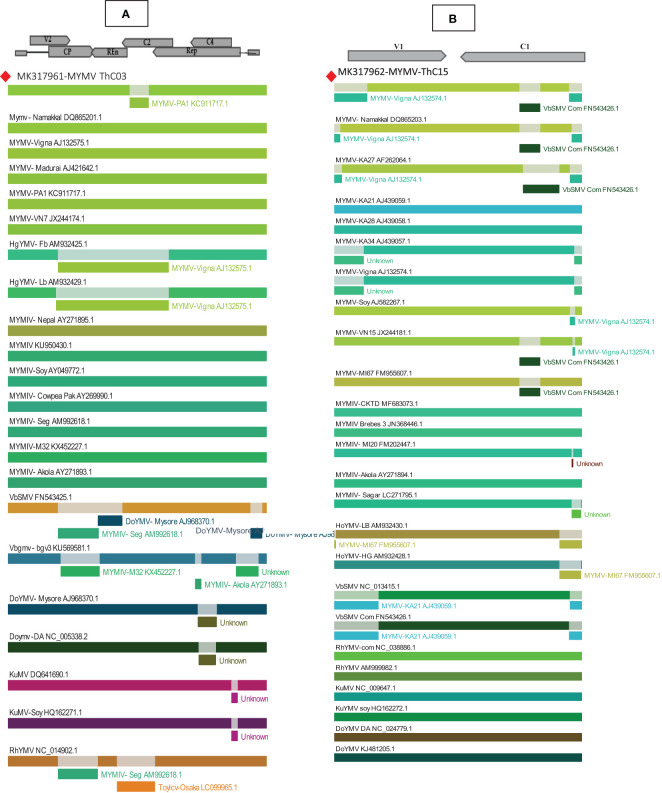
Recombination analyses of MYMV **(A)** MK317961 (DNA-A), **(B)** MK317962 (DNA-B) with other selected begomoviruses using RDP- 4.97. The top of the picture shows a linear genome map of begomoviruses, with arrows pointing in the direction of the genes to reveal recombinant segments.

Recombination analysis for isolate MYMV in the present study was performed using the Recombinant Detection Programme (RDP 4.97) to identify the potential recombinant parental isolates and recombinant breakpoints. In the RDP analysis, DNA-A (MYMV-MK31791-ThCo3) along with 25 New World and 23 Old World begomovirus nucleotide sequences and DNA-B (MK317961-MYMV-ThC15) with the 21 New World and 23 Old World begomovirus nucleotide sequences retrieved from the NCBI database from different host plants were analyzed. The RDP results for DNA-A showed that 15 recombination events were detected in the analysis. Among the 15 recombination events, 11 were detected by more than three algorithms. Interestingly, only one recombination event was detected for DNA-A (MK317961-MYMV-ThC03) using three recombination methods (GENECONV, BOOTSCAN, and SiScan), which is a single recombination event of MYMV DNA-A with recombinant breakpoints at nucleotide locations 1,436–1,584 on the genome of MYMV-ORF AC2 (transcription activation protein) in the present isolate. The present isolate contributed to the recombination event as a potential major parent identified as MYMV-PA1 (KC911717) and as a potential minor parent, MYMV-Namakkal (DQ86520). Importantly, none of the other MYMV isolates exhibited any recombination events. Compared with MYMV and MYMIV, the other begomoviruses were prone to recombination, with the recombination analysis detecting several events. The *p*-values of the identified recombination events were given in GENECONV (4.823× 10^−02^), BOOTSCAN (7.813× 10^−03^), and SiScan (7.466× 10^−03^). The same event was also detected in other MYMV isolates, including MYMV-Namakkal (DQ8652), MYMV-VN15 (JX244181), MYMV-KA27 (AF262064), and MYMV-M167 (FM955607) (**Figure 4A**).

Similarly, RDP analysis of the DNA-B component detected 43 recombination events. Of these, 23 events were detected by more than three algorithms, and the *p*-values were found to be significant. The isolates of DNA-B in the present study (MK317962-MYMV-ThC15) revealed the presence of two recombination events. The first recombination event pans from the nucleotide coordinates of 472 to 2,560 nt coded for the movement protein and the nuclear shuttle protein, including the ori in the stem and loop sequences. This event was found to be significant for all the algorithms employed. The *p*-values for every algorithm are given in **Table 1**. Interestingly, this event involved MYMV-Vigna (AJ132574) as a minor parent and MYMIV-M120 (FM202447) as a major parent. It is also significant that the same recombinational event was observed in the DNA-B components of four more MYMV isolates: MYMV-Namakkal (DQ8652.1), MYMV-VN15(JX244181), MYMV-KA27 (AF262064), and MYMV-Soy (AJ582267). The second recombination event was observed in the segment from 2,126 to 2,216 and was found to be significant only in the algorithms MAXCHI (*p* = 2.117× 10^−01^) and SISCAN (*p* = 8.045× 10^−04^). This recombination event included HgYMV-LB (AM932430.1) as a potential major parent and VbSMV-Com (FN543426.1) as a minor parent (**Figure 4B**).

**Table 1:** Recombination breakpoint and major and minor parental sequences detected in MYMV DNA-B.

Recombinant isolate	Recombinant Break points		Parental isolate		Recombination Detection methods^c^	P-value^d^
			Major^b^	Minor^a^		
	Begin	End				
**MK317962**	472	2560	MYMIV- M120 (Vigna) (FM202447)	MYMV-Vigna (AJ132574)	R	1.127 x 10^-08^
					G	6.857 x 10^-05^
					**B**	**5.985 x 10^-10^**
					M	6.220 x 10^-03^
					C	2.306 x 10^-0^
					S	4.632 x 10^-06^
					3Seq	8.903 x 10^-07^
**MK317962**	2126	2216	HgYMV-LB (AM932430.1)	VbSMV Com (FN543426.1)	M	2.117 X 10 ^-01^
					**S**	**8.045 x 10^-04^**

^a,b^ Minor and major parental refer to isolates contributing the smaller and larger fractions of the recombinant’s sequence, respectively. c, d Recombination detection methods and probability values, respectively.

### 3.4 Infectivity assay of MYMV

**Figure 5:**
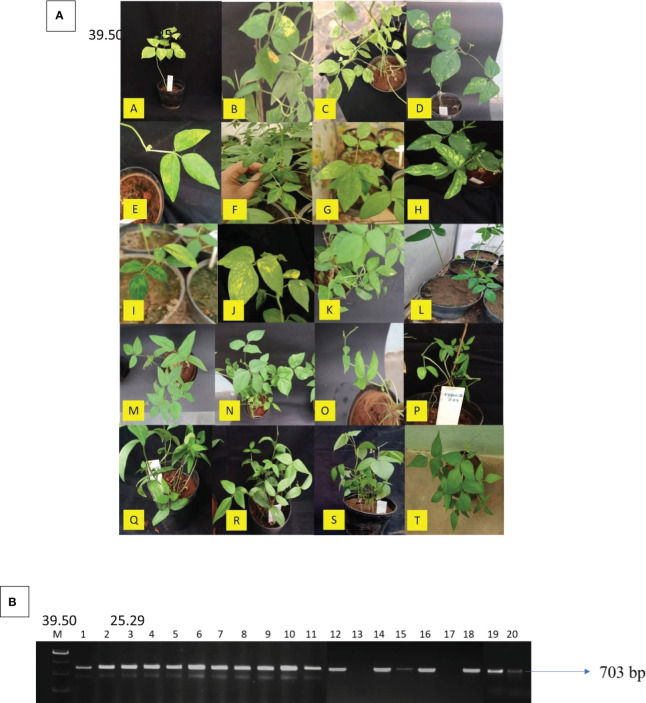
Infectivity and confirmation of cloned viral genomic component on mung bean cultivars **(A)** The symptomatic mung bean plants that were inoculated with agroinfectious constructs A. VRM(Gg)1, B. PUSA-101, C. PUSA-118, D. Maduramoong, E. VBN (Gg)2, F. PUSA-9871, G. LM 469, H. PLS-316, I. Bapatla, J. AVT/RMI-6/1, K. NDM 5-3, L. SML119, M. CO 8, N. CO7, O. CO 980, P. VGGRU2, Q. VGGRU3, R. VGGRU4, S. VGGRU1, T. PUSA – 0672, **(B)** Confirmation of MYMV resistance through MYMV coat protein primer on symptomatic B 39.50 25.29 A 39.50 25.29 plants, Lane M – 1kb marker, 1. VGGRU2, 2. VGGRU3, 3. VGGRU4, 4. CO8, 5. CO7, 6. CO980, 7. PUSA-118, 8. PUSA-9871, 9. VBN (Gg)2, 10. PLS- 316, 11. LM 469, 12. NDM 5-3, 13. VGGRU1, 14. AVT/RMI-6/1, 15. Maduramoong, 16. SML119, 17. PUSA-0672, 18. PUSA-101, 19. Bapatla, 20. VRM(Gg)1 (in this figure different gels are merged together as the gels contained many unpublished mung bean lines, the results for the cultivar in the present study were only shown and the original gels attached in **Supplementary Figure** (BI,BII,BIII).

To assess the infectivity of the MYMV isolates, agroinfectious constructs of both genomes were introduced into the overnight-sown seeds of 20 mung bean cultivars [i.e., VGGRU2, VGGRU3, VGGRU4, CO8, CO7, CO980, PUSA-118, PUSA-9871, VBN (Gg)2, PLS-316, LM469, Bapatla, VGGRU1, NDM 5–3, AVT/RMI-6/1, Maduramoong, SML119, PUSA-0672, PUSA-101, and VRM(Gg)1] using the agroinoculation technique. When both genomic components (DNA-A + DNA-B) were co-inoculated, typical yellow mosaic symptoms were observed in mung bean at 15–20 days post-inoculation (dpi). Among the tested cultivars, VGGRU1 and PUSA-0672 were found to be resistant; VGGRU2, VGGRU3, and VGGRU4 were found to be moderately susceptible; CO8, CO7, and CO980 were found to be susceptible; and PUSA-118, PUSA-9871, PUSA-101, PLS-316, LM469, Bapatla, NDM 5–3, AVT/RMI-6/1, Maduramoong, SML119, VRM (Gg)1, and VBN (Gg)2 were found to be highly susceptible (**Figure 5A**). The DNA-A or DNA-B genomic components individually failed to induce disease symptoms. As expected, the empty vector pCAMBIA2300 (negative control) induced no disease symptoms in the test plants even after 180 dpi. The symptomatic plants were confirmed for the amplification of MYMV using PCR, with amplification on the CP region (703 bp) confirming the presence of MYMV; the resistant plants were observed with no amplification product (**Figure 5B**).

## 4 Discussion

Begomoviruses affect agricultural and horticultural crops, which include more than 300 species, and cause an estimated yield loss of as high as 100% (Nagendran et al., 2016; Zerbini et al., 2017). MYMV and MYMIV are known to cause YMD in various leguminous and non-leguminous crops (Usharani et al., 2004; Qazi et al., 2006; Biswas et al., 2008; Akhtar et al., 2011; Naimuddin et al., 2011; Shahid et al., 2012; Agnihotri et al., 2019). Madhumitha et al. (2019) surveyed the incidence of YMD in mung bean-growing areas of southern India (Coimbatore, Tamil Nadu, India) and observed severe YMD symptoms. A novel YMV isolate was identified in the survey, which was characterized by RCA (Inoue-Nagata et al., 2004), cloning, and sequencing. RCA by *phi*29 DNA polymerase offers an exclusive perspective to study the range of circular molecules of viral origin. John et al. (2008) suggested that RCA provides the opportunity to generate full genome-sized DNA fragments of the viruses for their characterization by utilizing *phi*29 DNA polymerase. The RCA of the YMD-infected mung bean sample from southern India identified two DNA molecules: DNA-A (2,726 bp) and DNA-B (2,679 bp). The sequence analysis revealed that the present isolate has the corresponding ORFs depicting an Old World begomovirus. Two genes are present in the virion sense (AV1 and AV2) and four in the complementary sense (AC1, AC2, AC3, and AC4) (Padidam et al., 1999; Paximadis et al., 1999; Qazi et al., 2007; Kamaal et al., 2015; Agnihotri et al., 2019; Ansar et al., 2021). The characteristic feature of the genome organization in the present study indicated that the presence of ORF AV2 in DNA-A was reported as absent in New World begomoviruses (Rybicki, 1994; Stanley et al., 2005). Interestingly, the species observed as MYMV based on their nomenclature were found to be associated with the findings of Brown et al. (2015). In general, begomoviruses of a geographical origin are grouped together due to their high degree of conservation in the coat protein region, which is required for the recognition and transmission of the virus by the whitefly genotype, which is prevalent in that region. This rule of thumb is not followed for legumoviruses (begomoviruses, especially those infecting leguminous crop species). However, the same whitefly genotypes actively transmit them and are not grouped with other begomoviruses (Fauquet et al., 2005). Exceptionally, within the legumoviruses, MYMV and MYMIV cluster together, and DoYMV occupies a separate clade (Bag et al., 2014; Akram et al., 2015; Malathi et al., 2017). The phylogenetic analysis of DNA-A and DNA-B confirmed that MYMV clustered with the other Old World legumoviruses and showed distinct variation from the cluster of New World begomoviruses.

In the present study, a major recombination event was identified *in silico* in the whole DNA-B, for which MYMIV-M120 (FM202447) and MYMV-Vigna (AJ132574) were identified as the event’s major and minor parents, respectively, contributing to the inverted repeat (IR) region. This clearly indicates that the DNA-B of the MYMV-ThC15-India study is a recombinant, with the majority of the sequence derived from MYMV with a CR from MYMIV. This component exchange process, also known as pseudo-recombination for begomoviruses, has been reported for the recombinant DNA-B from India (Karthikeyan et al., 2004; John et al., 2008; Banerjee et al., 2018; Akram et al., 2020; Chowdary et al., 2022). Directly repeated sequences or short iterated elements called iterons, that are crucial to replication are present close to the TATA box and are crucial *cis*-elements in the ori that facilitate template recognition (Arguello-Astorga et al., 1994; Fontes et al., 1994). The putative Rep-binding iteron sequence, which consists of eight nucleotide repeats, was identified as ATCGGTGT, which occurs as one copy in the 5′ of the CR and as two tandem repeats upstream of the TATA box in DNA-A. In the case of DNA-B, one of the tandem repeats of the iteron is ATTGGTGT. However, DNA-B may have undergone recombination to circumvent the incompatibility between the iteron and the Rep proteins in the CR (Chowdary et al., 2022).

In disease resistance breeding programs, visual monitoring of the symptoms that occur on the plant, primarily after infection, is the main method of determining MYMV resistance. As MYMV symptoms may not always be present in the field, it can be challenging to identify the true resistant lines. These can be easily overcome by using the agroinoculation method. The primary advantage of agroinoculation is that it causes homogeneous disease symptoms that are simpler to measure than those of a natural infection (Sudha et al., 2015). To verify the infectivity and to determine the virus-resistant lines in the current study, agroinoculation screening was carried out by incubating the *Agrobacterium* cells at 28°C with a cell density of 1 (at OD_600_), with the maximum infectivity of 100% being achieved. The agroinoculated samples, by co-delivering MYMV DNA-A and DNA-B, showed more efficacy in the identification of resistant sources compared with field screening, as reported by various researchers (Jacob et al., 2003; Usharani et al., 2004; Karthikeyan et al., 2011; Sudha et al., 2013; Bag et al., 2014; Madhumitha et al., 2022).

## 5 Conclusions

In future applications, breeders and plant pathologists can augment their screening techniques beyond reliance on whitefly-mediated assays by employing the agroinoculation method. This strategy presents a robust avenue for distinguishing between cultivars exhibiting resistance and susceptibility to YMD. This approach offers promising prospects, providing a reliable means to identify plant resistance, thereby enhancing breeding and disease management efforts.

## Data availability statement

The original contributions presented in the study are included in the article/**Supplementary Material**. Further inquiries can be directed to the corresponding authors.

## Author contributions

MB: Investigation, Methodology, Writing – original draft. TT: Formal analysis, Writing – review & editing. KA: Methodology, Writing – review & editing. SR: Writing – review & editing. VR: Writing – review & editing. MoS: Supervision, Writing – review & editing. SN: Writing – review & editing. SK: Conceptualization, Methodology, Writing – review & editing. RM: Investigation, Project administration, Resources, Methodology, Writing – review & editing. SM: Investigation, Supervision, Methodology, Writing – review & editing.
