# Supplementary material for: Unveiling mungbean yellow mosaic virus: molecular insights and infectivity validation in mung bean (Vigna radiata) via infectious clones
Source: Front Plant Sci. 2024 Aug 2;15:1401526. doi: 10.3389/fpls.2024.1401526 (PMC11327075; doi:10.3389/fpls.2024.1401526)
Supplement: Supplementary file 1 [file Table_1.docx]

**Table S1** Genome organization of MYMV DNA-A (MK317961-MYMV-ThC03)

| **Features** | **AC1** | **AC2** | **AC3** | **AC4** | **AV1** | **AV2** |
| --- | --- | --- | --- | --- | --- | --- |
| **Start codon - Stop codon** | 1524 – 2612 nt | 1624 – 1217 nt | 1476 – 1072 nt | 2461 – 2162 nt | 302 – 1075 nt | 142 – 492 nt |
| **Gene** | Replication intiation protein gene | Transcription activator protein gene | Replication enhancer protein gene | Symptom determinant protein | Coat protein gene | Pre coat protein gene |
| **Molecular weight (kDa)** | ~40 kDa | ~15 kDa | ~15 kDa | ~11kDa | ~28 kDa | ~13 kDa |
| **Number of amino acids** | 362 | 135 | 134 | 99 | 257 | 116 |
| **Predicted function** | Replication initiate of RCA | PTGS suppressor and transcriptional activator of viral sense strand | Enhancement of replication | PTGS suppressor and expression of symptoms | Encapsidate and regulate of ssDNA | PTGS suppressor movement of viral genome into nucleus in monopartite viruses |
