# Supplementary material for: Unveiling mungbean yellow mosaic virus: molecular insights and infectivity validation in mung bean (Vigna radiata) via infectious clones
Source: Front Plant Sci. 2024 Aug 2;15:1401526. doi: 10.3389/fpls.2024.1401526 (PMC11327075; doi:10.3389/fpls.2024.1401526)
Supplement: Supplementary file 2 [file Table_2.docx]

**Table S2** Genome organization of MYMV DNA-B (MK317962-MYMV-ThC15)

| **Features** | **BC1** | **BV1** |
| --- | --- | --- |
| **Start codon – Stop codon** | 1219 -2115 nt | 423 -1193 nt |
| **Gene** | Movement protein gene | Nuclear shuttle protein gene |
| **Molecular weight (K Da)** | ~28 kDa | ~33kDa |
| **Number of amino acids** | 256 | 298 |
| **Predicted function** | Movement of viral genome | Shuttles viral genome from nucleus into plasma membrane |
