# Supplementary material for: Unveiling mungbean yellow mosaic virus: molecular insights and infectivity validation in mung bean (Vigna radiata) via infectious clones
Source: Front Plant Sci. 2024 Aug 2;15:1401526. doi: 10.3389/fpls.2024.1401526 (PMC11327075; doi:10.3389/fpls.2024.1401526)
Supplement: Supplementary file 3 [file Table_3.docx]

**Table S3** The pairwise identities of nucleotide sequence of DNA-A (MK317961-MYMV-ThC03) and other NCBI retrieved Genbank begomovirus genomes

| **Virus** | **Accession number** | **Identity (%)** | **Host** | **Country** |
| --- | --- | --- | --- | --- |
| *Mungbean yellow mosaic virus* | MW736047 | 98.30 | Mung bean | India |
| *Mungbean yellow mosaic virus* | MW736048 | 98.20 | Mung bean | India |
| *Mungbean yellow mosaic virus* | MW736045 | 98.20 | Mung bean | India |
| *Mungbean yellow mosaic virus* | DQ865201 | 98.20 | Moth bean | India |
| *Mungbean yellow mosaic India virus* | MN698289 | 83.20 | Moth bean | India |
| *Mungbean yellow mosaic India virus* | MN885468 | 83.50 | Soybean | Pakistan |
| *Mungbean yellow mosaic India virus* | EU523045 | 80.80 | Soybean | India |
| *Rhynchosia yellow mosaic virus* | AM999981 | 74.60 | Jumby bean | Pakistan |
| *Rhynchosia yellow mosaic virus* | FM208847 | 74.60 | Jumby bean | Pakistan |
| *Rhynchosia yellow mosaic virus* | KP752090 | 74.00 | French bean | India |
| *Rhynchosia yellow mosaic virus* | GQ472985 | 58.80 | Soybean | Nigeria |
| *Soybean chlorotic blotch virus* | GQ472987 | 58.80 | Butterfly Pea | Nigeria |
| *Soybean chlorotic blotch virus* | KC508643 | 58.30 | Lima bean | Nigeria |
| *Horsegram yellow mosaic virus* | AJ627904 | 84.20 | Horse gram | India |
| *Horsegram yellow mosaic virus* | OP784475 | 84.70 | Horse gram | India |
| *Horsegram yellow mosaic virus* | OP777488 | 84.60 | Horse gram | India |
| *Kudzu mosaic virus* | DQ641690 | 69.30 | Kudzu | Vietnam |
| *Kudzu mosaic virus* | ON181435 | 68.90 | Kudzu | China |
| *Kudzu mosaic virus* | MW805421 | 68.60 | Kudzu | China |
| *Dolichos yellow mosaic virus* | MH795972 | 61.60 | Dolichos bean | India |
| *Dolichos yellow mosaic virus* | AM157412 | 61.90 | Dolichos bean | India |
| *Dolichos yellow mosaic virus* | KJ481204 | 62.30 | Dolichos bean | India |
| *Soybean mild mottle virus* | GQ472984 | 61.90 | Soybean | Nigeria |
| *Bean calico mosaic virus* | AF110189 | 52.90 | Bean | USA |
| *Bean yellow mosaic Mexico virus* | FJ944023 | 55.20 | Bean | Mexico |
| *Bean white chlorosis mosaic virus* | JN848772 | 54.20 | Bean | Venezuela |
| *Bean leaf crumple virus* | KX857725 | 53.50 | Bean | Colombia |
| *Bean latent virus* | MN158325 | 56.20 | Bean | Mexico |
| *Bean golden yellow mosaic virus* | L01635 | 54.40 | Bean | Dominican Republic |
| *Bean golden yellow mosaic virus* | MK241786 | 55.10 | Bean | Mexico |
| *Bean golden yellow mosaic virus* | MG659315 | 55.30 | Bean | Mexico |
| *Bean dwarf mosaic virus* | M88179 | 55.10 | Bean | Colombia |
| *Bean chlorosis virus* | JN848770 | 56.30 | Bean | Venezuela |
| *Bean golden mosaic virus* | M88686 | 55.80 | Bean | Brazil |
| *Bean golden mosaic virus* | KJ939776 | 56.80 | Phasey bean | Brazil |
| *Bean golden mosaic virus* | KJ939767 | 56.90 | Phasey bean | Brazil |
| *Common bean mottle virus* | KX011473 | 57.30 | Bean | Cuba |
| *Common bean severe mosaic virus* | KX011475 | 58.10 | Bean | Cuba |
| *Common bean severe mosaic virus* | KX011477 | 58.30 | Bean | Cuba |
| *Common bean severe mosaic virus* | KX011476 | 58.20 | Bean | Cuba |
| *Rhynchosia rugose golden mosaic virus* | HM236370 | 55.40 | Jumby bean | Cuba |
| *Rhynchosia mild mosaic virus* | FJ944019 | 58.10 | Jumby bean | Puerto Rico |
| *Rhynchosia golden mosaic virus* | DQ347950 | 56.70 | Jumby bean | Mexico |
| *Rhynchosia golden mosaic virus* | MK634355 | 56.90 | Jumby bean | Mexico |
| *Rhynchosia golden mosaic virus* | EU339939 | 56.70 | Asteraceae | Mexico |
| *Soybean blistering mosaic virus* | EF016486 | 54.70 | Soybean | Argentina |
| *Soybean blistering mosaic virus* | MN508210 | 56.60 | Bell Pepper | Argentina |
| *Soybean blistering mosaic virus* | MN486865 | 56.50 | Bell Pepper | Argentina |
