# Supplementary material for: Unveiling mungbean yellow mosaic virus: molecular insights and infectivity validation in mung bean (Vigna radiata) via infectious clones
Source: Front Plant Sci. 2024 Aug 2;15:1401526. doi: 10.3389/fpls.2024.1401526 (PMC11327075; doi:10.3389/fpls.2024.1401526)
Supplement: Supplementary file 4 [file Table_4.docx]

**Table S4** The pairwise identities of amino acid sequence of isolate DNA-A (MK317961-MYMV-ThC03) and other NCBI retrieved Genbank begomovirus genomes

| **S. No.** |  | **Genbank**  **Accession Number** | **Virus isolates and Country** | **Host** | **AC1**  **(%)** | **AC2 (%)** | **AC3**  **(%)** | **AC4**  **(%)** | **AV1**  **(%)** | **AV2**  **(%)** |
| --- | --- | --- | --- | --- | --- | --- | --- | --- | --- | --- |
|  | **Old world begomovirus** | MW736047 | MYMV-NaMu2-India | Mung bean | 97.20 | 56.0 | 71.0 | 97.90 | 99.60 | 100.00 |
|  |  | MW736048 | MYMV-NaMu1-India | Mung bean | 96.60 | 63.0 | 71.0 | 97.90 | 99.20 | 100.00 |
|  |  | MW736045 | MYMV-NaMu3-India | Mung bean | 97.50 | 56.0 | 71.0 | 98.90 | 99.20 | 100.00 |
|  |  | DQ865201 | MYMV-Na06-India | Moth bean | 98.30 | 94.80 | 90.20 | 96.90 | 100.0 | 100.00 |
|  |  | MN885468 | MYMIV-SR13-Pakistan | Soybean | 85.0 | 83.30 | 90.20 | 72.70 | 87.50 | 77.50 |
|  |  | OP784475 | HgYMV-CH-India | Horse gram | 85.0 | 85.10 | 84.30 | 75.70 | 91.80 | 76.70 |
|  |  | OP777488 | HgYMV-KD-India | Horse gram | 87.0 | 85.10 | 85.0 | 74.70 | 91.80 | 76.70 |
|  |  | EU523045 | MYMIV-Delhi-India | Soybean | 84.20 | 81.60 | 76.10 | 74.70 | 85.20 | 69.80 |
|  |  | AJ627904 | HgYMV-Madurai-India | Horse gram | 84.20 | 84.40 | 83.50 | 75.70 | 91.40 | 75.80 |
|  |  | MN698289 | MYMIV-BelgaumMol-India | Moth bean | 83.90 | 72.60 | 73.80 | 74.70 | 91.40 | 78.40 |
|  |  | AM999981 | RhYMV-Lah-Pakistan | Jumby bean | 79.90 | 60.80 | 53.60 | 67.60 | 85.60 | 52.40 |
|  |  | FM208847 | RhYMV-MI32- Pakistan | Jumby bean | 79.90 | 60.80 | 53.60 | 67.60 | 85.60 | 36.00 |
|  |  | KP752090 | RhYMV-pBdGn05-India | Jumby bean | 77.50 | 59.70 | 55.10 | 68.60 | 85.60 | 50.0 |
|  |  | DQ641690 | KuMV-Australia | Kudzu | 70.10 | 58.90 | 59.40 | 39.30 | 79.70 | 41.80 |
|  |  | ON181435 | KuMV-CQ-China | Kudzu | 69.60 | 56.80 | 59.40 | 42.10 | 80.50 | 42.60 |
|  |  | MW805421 | KuMV-YG01-China | Kudzu | 69.60 | 56.80 | 59.40 | 37.30 | 80.50 | 41.80 |
|  |  | GQ472987 | SbCBV-Cs1-Nigeria | Butterfly Pea | 66.50 | 43.80 | 37.60 | 33.30 | 67.70 | - |
|  |  | KC508643 | SbCBV-V7-Denmark | Lima bean | 66.50 | 43.80 | 37.60 | 32.30 | 68.00 | - |
|  |  | KJ481204 | DoYMV-DA-India | Dolichos bean | 60.50 | 53.50 | 55.10 | 24.20 | 80.10 | - |
|  |  | MH795972 | DoYMV-TNTM1-India | Dolichos bean | 59.50 | 52.80 | 56.60 | 24.20 | 74.30 | - |
|  |  | AM157412 | DoYMV-BAN1-India | Dolichos bean | 59.70 | 52.80 | 56.60 | 24.20 | 78.20 | - |
|  |  | GQ472985 | SbCBV-Sb19-Nigeria | Soybean | 66.50 | 43.80 | 37.60 | 33.33 | 67.70 | - |
|  |  |  |  | **Mean** | **78.40** | **63.92** | **64.44** | **60.63** | **85.03** | **50.89** |
|  |  |  |  |  |  |  |  |  |  |  |
|  | **New world begomovirus** | MN508210 | SbBMV-ARSP317-Argentina | Bell Pepper | 61.90 | 49.20 | 37.50 | 38.30 | 73.10 | - |
|  |  | MN486865 | SbBMV-ARSP145-Argentina | Bell Pepper | 61.90 | 48.50 | 36.70 | 37.30 | 73.10 | - |
|  |  | FJ944019 | RhMMV-PR79-Puerto Rico | Jumby bean | 67.40 | 44.60 | 44.10 | - | 70.80 | - |
|  |  | KX011476 | CBSMV-CuMa96-Cuba | Bean | 66.90 | 46.80 | 38.90 | 43.40 | 71.20 | - |
|  |  | MK634355 | RhGMV-M02-Mexico | Jumby bean | 66.90 | 43.20 | 43.30 | 44.40 | 72.30 | - |
|  |  | KX011473 | CBMoV-MA6-Cuba | Bean | 66.90 | 43.90 | 46.30 | 23.50 | 70.80 | - |
|  |  | KX011477 | CBSMV-CuMa99-Cuba | Bean | 66.60 | 51.0 | 41.90 | 43.40 | 71.50 | - |
|  |  | KX011475 | CBSMV-MA99-Cuba | Bean | 66.30 | 50.30 | 41.10 | 42.40 | 71.50 | - |
|  |  | HM236370 | RhRGMV-CA171-Cuba | Jumby bean | 62.10 | 47.0 | 36.0 | 37.30 | 72.70 | - |
|  |  | JN848770 | BChMV-BA459-Venezuela | Bean | 61.0 | 46.30 | 35.20 | 38.30 | 72.30 | - |
|  |  | FJ944023 | BYMMxV-11-Mexico | Bean | 60.60 | 47.70 | 35.20 | - | 72.30 | - |
|  |  | KJ939776 | BGMV-Flt11-Brazil | Phasey bean | 60.20 | 47.0 | 34.50 | 29.20 | 72.30 | - |
|  |  | KJ939767 | BGMV-Flt2-Brazil | Phasey bean | 60.20 | 47.0 | 34.50 | 29.20 | 72.30 | - |
|  |  | MK241786 | BGYMV-CN61-Mexico | Bean | 59.10 | 37.40 | 37.50 | - | 71.50 | - |
|  |  | MG659315 | BGYMV-CN25-Mexico | Bean | 59.40 | 37.40 | 37.50 | - | 71.50 | - |
|  |  | MN158325 | BLV-CN30-Mexico | Bean | 58.60 | 47.70 | 36.70 | 44.40 | 73.50 | - |
|  |  | JN848772 | BWCMV-Rub-Venezuela | Bean | 58.30 | 44.10 | 36.00 | 39.30 | - | - |
|  |  | KX857725 | BLCrV-HA-Colombia | Bean | 50.10 | 34.40 | 34.50 | 12.30 | - | - |
|  |  | AF110189 | BChV-C58-USA | Bean | 49.30 | 33.80 | 35.20 | 17.60 | - | - |
|  |  |  |  | **Mean** | **61.25** | **44.59** | **38.03** | **27.38** | **60.67** | **0.00** |
