# Supplementary material for: Unveiling mungbean yellow mosaic virus: molecular insights and infectivity validation in mung bean (Vigna radiata) via infectious clones
Source: Front Plant Sci. 2024 Aug 2;15:1401526. doi: 10.3389/fpls.2024.1401526 (PMC11327075; doi:10.3389/fpls.2024.1401526)
Supplement: Supplementary file 5 [file Table_5.docx]

**Table S5** The pairwise identities of nucleotide sequence of DNA-B (MK317962-MYMV-ThC15) and other NCBI retrieved Genbank begomovirus genomes

| **Virus** | **Accession number** | **Identity (%)** | **Host** | **Country** |
| --- | --- | --- | --- | --- |
| *Mungbean yellow mosaic virus* | DQ865203 | 98.10 | Moth bean | India |
| *Mungbean yellow mosaic virus* | AF262064 | 96.10 | Urd bean | India |
| *Mungbean yellow mosaic virus* | MN698276 | 95.40 | Mung bean | India |
| *Mungbean yellow mosaic virus* | MN698283 | 95.50 | Minni Payaru | India |
| *Mungbean yellow mosaic virus* | MN602420 | 95.80 | Mung bean | India |
| *Mungbean yellow mosaic India virus* | MN020536 | 70.20 | Mung bean | India |
| *Mungbean yellow mosaic India virus* | MW717979 | 70.30 | Urd bean | India |
| *Mungbean yellow mosaic India virus* | MF683073 | 70.50 | Tomato | India |
| *Mungbean yellow mosaic India virus* | JN368446 | 70.50 | Soybean | Indonesia |
| *Dolichos yellow mosaic virus* | KJ481205 | 49.80 | Dolichos bean | India |
| *Dolichos yellow mosaic virus* | MT108191 | 49.80 | Country bean | Bangladesh |
| *Kudzu mosaic virus* | DQ641691 | 48.40 | Kudzu | Vietnam |
| *Kudzu mosaic virus* | HQ162272 | 54.70 | Soybean | Vietnam |
| *Horsegram yellow mosaic virus* | AJ627905 | 53.90 | Horse gram | Vietnam |
| *Horsegram yellow mosaic virus* | AM932426 | 66.50 | French bean | India |
| *Horsegram yellow mosaic virus* | AM932428 | 65.70 | Horse gram | India |
| *Rhynchosia yellow mosaic virus* | AM999982 | 66.50 | Jumby bean | Pakistan |
| *Rhynchosia yellow mosaic virus* | FM208848 | 57.10 | Jumby bean | Pakistan |
| *Rhynchosia yellow mosaic virus* | KP752091 | 57.20 | French bean | India |
| *Soybean chlorotic blotch virus* | GQ472986 | 45.30 | Soybean | Nigeria |
| *Soybean chlorotic blotch virus* | KT444612 | 45.00 | Common Wireweed | Nigeria |
| *Rhynchosia rugose golden mosaic virus* | HM236371 | 36.50 | Jumby bean | Cuba |
| *Rhynchosia mild mosaic virus* | FJ944020 | 37.10 | Jumby bean | Puerto Rico |
| *Rhynchosia golden mosaic virus* | EU339937 | 34.40 | Soybean | Mexico |
| *Rhynchosia golden mosaic virus* | DQ356429 | 34.60 | Soybean | Mexico |
| *Rhynchosia golden mosaic virus* | MK634539 | 34.70 | Jumby bean | Mexico |
| *Bean calico mosaic virus* | AF110190 | 36.40 | Bean | Mexico |
| *Common bean severe mosaic virus* | KX096982 | 36.90 | Bean | Cuba |
| *Common bean severe mosaic virus* | KX096981 | 37.70 | Bean | Cuba |
| *Common bean mottle virus* | KX011474 | 38.10 | Bean | Cuba |
| *Bean golden mosaic virus* | M88687 | 33.90 | Bean | Brazil |
| *Bean golden mosaic virus* | MN822293 | 37.60 | *Macroptilium erythroloma* | Brazil |
| *Bean golden mosaic virus* | MT626961 | 37.90 | Lima bean | Brazil |
| *Bean chlorosis virus* | JN848771 | 36.70 | Bean | Venezuela |
| *Bean dwarf mosaic virus* | M88180 | 32.60 | Bean | Colombia |
| *Bean chlorotic mosaic virus* | JQ283246 | 36.70 | Bean | Venezuela |
| *Bean leaf crumple virus* | KX857726 | 36.50 | Bean | Colombia |
| *Bean leaf crumple virus* | OK044473 | 38.10 | Burgundy Bean | Venezuela |
| *Bean latent virus* | MN158326 | 37.40 | Bean | Mexico |
| *Bean golden yellow mosaic virus* | L01636 | 35.70 | Bean | Dominican Republic |
| *Bean golden yellow mosaic virus* | M91605 | 35.60 | Bean | Dominican Republic |
| *Bean golden yellow mosaic virus* | AF173556 | 35.50 | Bean | Mexico |
