# Supplementary material for: Unveiling mungbean yellow mosaic virus: molecular insights and infectivity validation in mung bean (Vigna radiata) via infectious clones
Source: Front Plant Sci. 2024 Aug 2;15:1401526. doi: 10.3389/fpls.2024.1401526 (PMC11327075; doi:10.3389/fpls.2024.1401526)
Supplement: Supplementary file 6 [file Table_6.docx]

**Table S6** The pairwise identities of amino acid sequence of DNA-B (MK317962-MYMV-ThC15) and other NCBI retrieved Genbank begomovirus genomes

| **S. No.** | **Genbank Accession number** | **Virus isolates and Country** | **Host** | **BC1**  **(%)** | **BV1**  **(%)** |
| --- | --- | --- | --- | --- | --- |
|  | DQ865203 | MYMV-Namakkal-India | Moth bean | 99.60 | 100 |
|  | AF262064 | MYMV-KA27-India | Urd bean | 99.30 | 98.40 |
|  | MN698276 | MYMV-Mu2-India | Mung bean | 99.60 | 100 |
|  | MN698283 | MYMV-RaVs-India | Minni Payaru | 100 | 98.80 |
|  | MN602420 | MYMV-DhMu1-India | Mung bean | 100 | 100 |
|  | MN020536 | MYMIV-MRHA2-India | Mung bean | 88.20 | 75.30 |
|  | MW717979 | MYMIV-Ur2-India | Urd bean | 89.20 | 76.10 |
|  | MF683073 | MYMIV-CKTD-India | Tomato | 88.90 | 76.10 |
|  | MW717969 | MYMIV-1NRF-India | Urd bean | 88.20 | 75.30 |
|  | JN368446 | MYMIV-Brebes3-Indonesia | Soybean | 87.90 | 76.50 |
|  | AJ627905 | HgYMV-Coimbatore-India | Horse gram | 85.20 | 73.80 |
|  | AM932428 | HgYMV-Banglore-India | Cowpea | 69.40 | 73.40 |
|  | AM932426 | HgYMV-Fb-India | French bean | 85.20 | 73.80 |
|  | KJ481205 | DoYMV-Kanpur DA-India | Dolichos bean | 69.40 | 52.80 |
|  | MT108191 | DoYMV-BP-India | Country bean | 68.70 | 51.30 |
|  | HQ162272 | KuMV-Hanoi2010-Vietnam | Soybean | 71.40 | 57.50 |
|  | FM208848 | RhYMV-MI34-Pakistan | Jumby bean | 78.10 | 57.80 |
|  | KP752091 | RhYMV-BdGn04-India | French bean | 68.10 | 57.0 |
|  | AM999982 | RhYMV-Lahore-Pakistan | Jumby bean | 77.80 | 58.20 |
|  | GQ472986 | SbCBV-Sb19-Nigeria | Soybean | 49.80 | 38.0 |
|  | DQ641691 | KuMV-Hoabinh-VietNam | Kudzu | 71.40 | 56.50 |
|  | KT444612 | SbCBV-IbSi-3614-Nigeria | Common Wireweed | 60.50 | 38.0 |
|  | KX096982 | CBSMV-Mayabeque99-Cuba | Bean | 39.0 | 27.10 |
|  | HM236371 | RhRGMV-Ca171-Cuba | Jumby bean | 39.70 | 26.40 |
|  | FJ944020 | RhMMV-PR79-Puerto Rico | Jumby bean | 39.40 | 26.0 |
|  | EU339937 | RhGMV-1068-Mexico | Soybean | 38.40 | 25.20 |
|  | DQ356429 | RhGMV-SO1045-Mexico | Soybean | 39.0 | 24.50 |
|  | MK634539 | RhGMV-Sol06-Mexico | Jumby bean | 39.0 | 25.20 |
|  | KX011474 | CBMoV-Mayabeque6-Cuba | Bean | 40.0 | 24.20 |
|  | JN848771 | BChMV-LaBa459-Venezuela | Bean | 38.10 | 23.80 |
|  | JQ283246 | BWCMV-Rubio932-Venezuela | Bean | 38.40 | 26.10 |
|  | KX857726 | BLCrV-HA-Colombia | Bean | 40.70 | 24.50 |
|  | AF110190 | BChV-Mexico | Bean | 40.30 | 25.40 |
|  | OK044473 | BLCrV-VE-V4-Venezuela | Burgundy Bean | 39.40 | 25.20 |
|  | MN158326 | BLV-NaCN30-Mexico | Bean | 38.70 | 23.30 |
|  | KX096981 | CBSMV-Mayabeque96-Cuba | Bean | 39.70 | 27.10 |
|  | MN822293 | BGMV-RDF327JB-Brazil | *Macroptilium erythroloma* | 40.70 | 25.0 |
|  | MT626961 | BGMV-BRPai133-Brazil | Lima bean | 40.70 | 25.40 |
|  | AF173556 | BGYMV-Chiapa-Mexico | Bean | 40.30 | 25.60 |
|  |  |  | **Mean** | **63.27** | **50.31** |
